# Colombian Sustainability Perspective on Fused Deposition Modeling Technology: Opportunity to Develop Recycled and Biobased 3D Printing Filaments

**DOI:** 10.3390/polym15030528

**Published:** 2023-01-19

**Authors:** Maria A. Morales, Alejandro Maranon, Camilo Hernandez, Veronique Michaud, Alicia Porras

**Affiliations:** 1 Grupo de Diseño de Productos y Procesos (GDPP), Department of Chemical and Food Engineering, Universidad de los Andes, Bogota 111711, Colombia; 2 Structural Integrity Research Group (GIE), Department of Mechanical Engineering, Universidad de los Andes, Bogota 111711, Colombia; 3 Sustainable Design in Mechanical Engineering Research Group (DSIM), Department of Mechanical Engineering, Escuela Colombiana de Ingenieria Julio Garavito, Bogota 111166, Colombia; 4 Laboratory for Processing for Advanced Composited (LPAC), École Polytechnique Fédérale de Lausanne (EPFL), Institute of Materials (IMX), CH-1015 Lausanne, Switzerland

**Keywords:** agro-industrial waste, circular economy, fused deposition modeling, plastics recycling

## Abstract

In the context of the preservation of natural resources, researchers show a growing interest in developing eco—friendly materials based on recycled polymers and natural fiber biocomposites to minimize plastic and agroindustrial waste pollution. The development of new materials must be integrated within the circular economy concepts to guarantee sustainable production. In parallel, fused deposition modeling, an additive manufacturing technology, provides the opportunity to use these new materials in an efficient and sustainable manner. This review presents the context of plastics and agro-industrial fiber pollution, followed by the opportunity to give them added value by applying circular economy concepts and implementing these residues to develop new materials for the manufacture of fused deposition modeling 3D printing technique feedstock. Colombian perspective is highlighted since 3D printing technology is growing there, and Colombian biodiversity represents a high reservoir of materials. Also, recycling in Colombia promotes compliance with the 2030 Agenda and the Sustainable Development Goals.

## 1. Introduction

**Figure 1:**
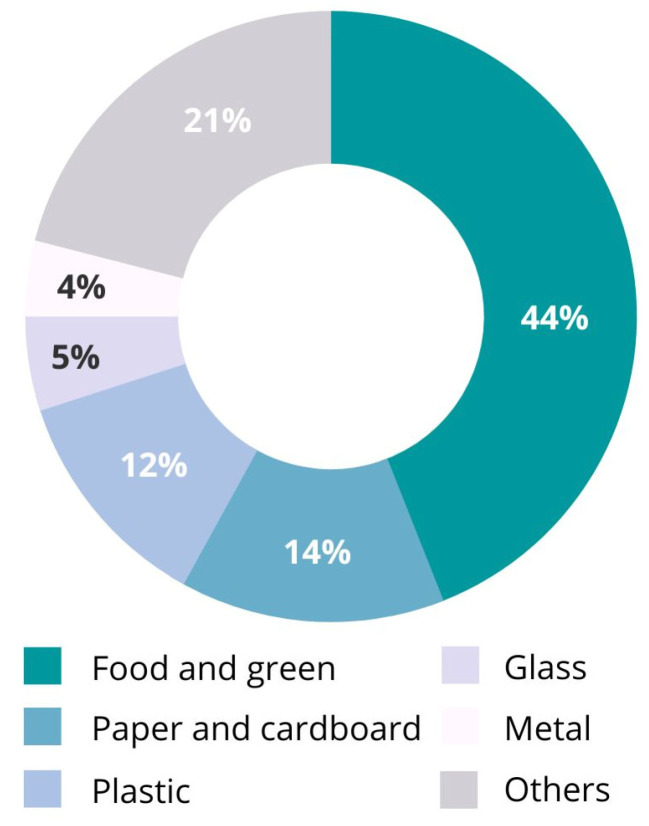
Global waste composition, by weight [2].

Since early times, humans have used and disposed of different natural resources. Nevertheless, waste disposal was not important due to the low population density and the high availability of land for waste. The problem began with the first human communities when waste generation became a consequence of life, urbanization, and economic development. One of the most important waste streams is municipal solid waste (MSW). It is estimated that, globally, 2.01 billion tons of MSW are generated annually, a number which is expected to grow to 3.40 billion tons by 2050 [1]. A standard waste audit determines the categorization of types of materials in MSW. Although waste production differs from country to country, the main categories are food and biological waste that can be broken down into compost, paper and cardboard, plastic, glass, metal, wood, and rubber and leather waste (Figure 1) [2].

Plastics represent the third most important MSW and are one of the most challenging materials concerning environmental pollution. Over the last 50 years, plastics have been increasingly used in various daily life products due to their versatility, low cost, and durability: in 2019, the global plastic production reached 370 million tons [3], and this amount is expected to increase to 900 million tons by 2050 [4]. Landfill, incineration, and recycling are the main strategies to manage plastic waste [5]. Nevertheless, large quantities of harmful compounds and greenhouse gases are released into the atmosphere during incineration, and plastic waste in landfills generates the loss of natural ecosystems.

Worldwide, 9% of plastic waste is recycled, 19% is incinerated, 50% is deposed in landfills, and 22% remains fully unattended [6]. In the case of Colombia, the plastic production capacity was 1.36 million metric tons in 2020 [7], and the recycling rate stands at 8.7%.

**Figure 2:**
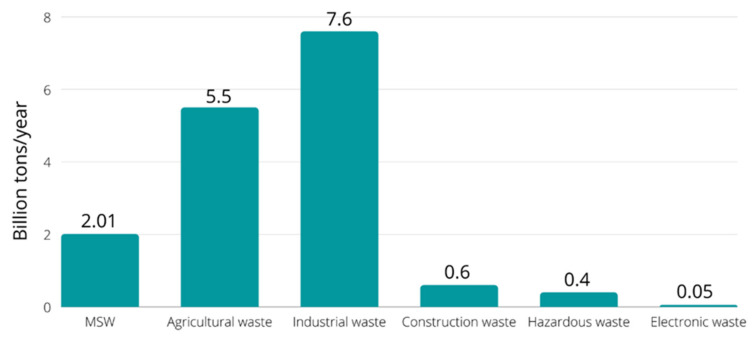
The global average of different steams waste generation [2,8,9,10].

Even though MSW is one of several waste streams countries must manage, agricultural waste, industrial waste, construction and demolition waste, hazardous waste, and medical waste can generate higher residues than MSW [2]. A global generation of those different waste streams is shown in Figure 2.

It is estimated that about 5.5 billion tons of agricultural waste are globally generated per year [11]. In Colombia, food supply centers and agricultural products processing (agro-industrial industry) bring about 7 million tons of residual biomass annually [12]. In general, agro-industrial waste is untreated and underutilized and ends up disposed into the soil, where biomass-induced microflora stimulates the production and emission of greenhouse gases and N_2_O, which have greater global warming potential than CO_2_. In addition, its incineration contributes to about 18% of total CO_2_ global emissions [13].

Plastic and agro-industrial sectors, among others, tend to follow a linear resource consumption model, where raw materials are collected, then transformed into products that are used until they are discarded as waste [14]. The disadvantages of such linearity could be seen in the product life cycle. Collecting raw materials and product production leads to high energy and water consumption, toxic substance emissions, and natural capital disruption. When these products are discarded, space is taken from natural areas, and harmful emissions are also generated [15]. Growing environmental awareness and increasing interest in sustainability have led industries to adopt responsible production and consumption methods. Those methods must be aligned with the emerging circular economy (CE). One of the principles is to design for durability, reuse, remanufacturing, and recycling to keep products, components, and materials circulating in the economy, minimizing produced waste [16]. Accordingly, there has been a great interest in using recycled and biobased materials to manufacture cost-competitive and biodegradable consumer goods [17,18]. 

Further, recent technologies are driving the transition to a CE. One industrial process well-suited to reusing post-consumer plastic is 3D printing, also known as additive manufacturing (AM) [19]. Additive manufacturing provides versatility in a wide range of industries and applications. It is considered environmentally friendly due to the reduced use of raw materials and has multiple possibilities to create complex geometry pieces, which offers freedom for design and innovation [20]. Worldwide its use reached an estimated US $15 trillion in 2020, with a forecasted growth of 24% for the next few years. In Colombia, according to the Observatorio de Economía Digital de Colombia, in 2018, only 6.8% of the companies adopted this technology [21].

The adoption of 3D printing technologies is essential for future industry competitiveness and environmental sustainability due to its advantages which have environmental benefits and enables mass production of low-demand products. Additionally, 3D printing increases the lifecycle of products that lose their functionality due to the lack of availability of spare parts that are not economically viable, enables global commercialization by facilitating production close to the place of consumption, and makes the production of personalized and unique products economically viable [22]. Furthermore, new materials implementation reduces the use of petroleum materials by combining them with bio-based materials to produce composites.

Thus, this review article explores the actual status of plastic and agroindustrial waste pollution, highlighting the importance of applying circular economy principles to keep materials in use. Also, data is collected about the tendencies in Fused Deposition Modeling (FDM), where recycled plastics and agro-industrial waste have been used as 3D printing feedstock. Our focus is presented in a national context to evaluate the innovation of this field in Colombia and the potential to develop biocomposite materials with local residues.

## 2. From a Linear to a Circular Economy: Waste Valorization

### 2.1. Plastic Pollution

Plastic pollution is one of the most significant environmental threats nature and humans face due to its accumulation in natural environments and landfills. The durability of plastics, and characteristics such as a wide range of operating temperatures, high thermal/electrical insulation, corrosion- and light- resistance, and sufficient mechanical properties (high strength-to-weight ratio, stiffness, toughness, and ductility) [23], show their potential to be used in a range of low-cost, low-weight, high-performance products [24]. 

Consequently, the production of plastics has increased over the last years; just in 2019, global plastic production reached 370 million tons [3]. Nevertheless, products and parts made of plastics have short life cycles and are replaced frequently or obsolesce over a short time, ending up in landfills, burned, or stocked in the environment [19,25]. In landfills, plastic debris produces a source of secondary environmental pollutants [26]. During the degradation process, plastics brittleness increases and fragmentation takes place until the achievement of lower molecular weight for further metabolism by microbes [27]. Microplastics can be consumed by marine organisms, contaminating the food chain. Also, they can contribute to greenhouse emissions and cause climate change [28].

**Figure 3:**
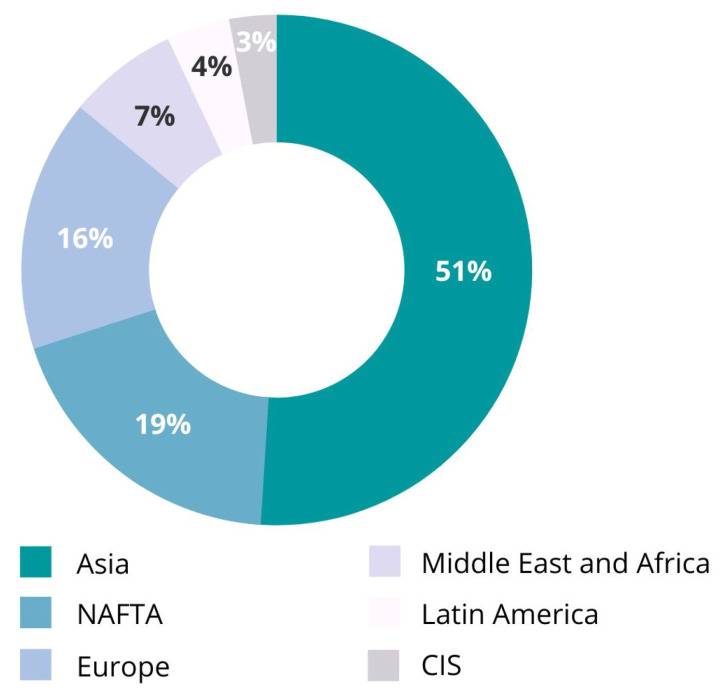
Distribution of global plastics production [3].

Figure 3 shows the distribution of global plastic production. Latin America encompasses 4% of global virgin plastics production, about 14.4 million tons in 2018 (United Nations Environment Programme) [29]. The Latin American region has the third highest waste generation per person, with a rate of 0.99 kg per person and per day [30]. This general rate is higher than the global average of 0.74 kg per person per day. Specifically, in Colombia, in 2018, waste generation was about 0.64 kg per day per person, among which 10.8% plastic residues [30]. The most significant volumes of production capacity correspond to polypropylene (PP) and polyvinyl chloride (PVC) plants, representing 73% of the total [7]. 

Further, Colombian plastic consumption is about 1,2 million tons per year. [31]. Nevertheless, because Colombia’s waste management infrastructure is still under development, only 17% of total solid waste is recycled [32], inducing pollution of ecosystems such as oceans, rivers, and mangroves. As a result, 90% of Atlantic coast beaches are contaminated with microplastics, and the Amazon and Magdalena are among the 20 most plastic-polluted rivers [33]. These levels of environmental pollution pose a threat to hundreds of species, and even to humans, due to the exposure to plastics entanglement, ingestion, and smothering that can produce reduced mobility, increased energy expenditure, reduced energy intake, injuries, and associated infections [34].

### 2.2. Agro-Industrial Waste

**Figure 4:**
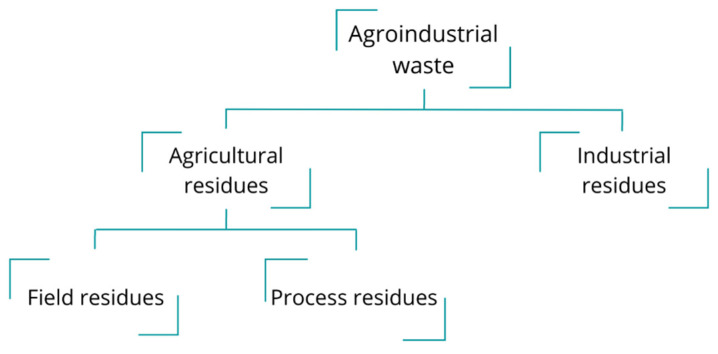
Classification of agro-industrial.

An additional massive source of pollution is agro-industrial waste, which was declared the third largest source of pond, lake, and reservoir pollution. Agriculture has provided society with essential products for thousands of years, making it the largest economic sector in many countries [35]. However, agricultural-based industries produce many residues from primary products and their industrialization every year. Agro-industrial waste is generated by the manufacturing and processing of agro-industrial products and can be categorized into two types of residues: Agricultural and industrial waste (Figure 4). Agricultural waste is defined as unwanted residues produced in agricultural activities and is divided into two categories: field residues and process residues. Field residues are materials left on the cultivated land after crop harvesting (e.g., stems, leaves, seed pods), and process residues are wastes obtained during and after the crop processing into value products (e.g., husks, seeds, roots, bagasse, straws). On the other hand, industrial residues are mainly produced during food and beverage processing waste (e.g., fruit and vegetable peels, meat waste, brewery wastewater, and oil residues [36,37].

In addition, according to the Food and Agriculture Organization of the United Nations, one-third of food produced globally (1.3 billion tons) is thrown away yearly [38]. Most of these waste products are untreated or underutilized, generating their uncontrolled incineration and deposition in landfills [39,40]. Continuously burning can reduce soil quality and make land more susceptible to erosion, and the smoke contributes to increasing carbon dioxide levels in the atmosphere, affecting greenhouse gas build-up [41].

Further, the most common chemical contaminant found in groundwater worldwide is nitrate from farming, which is responsible for discharging large quantities of organic matter, sediments, and saline solutions into water bodies. According to the National Summary of Assessed Waters Report in 2010, approximately 53% of global rivers and streams have been declared unfit for designed use [42].

In Latin America, countries such as Colombia, Ecuador, Paraguay, Mexico, and Panama are major agro-industrial waste producers due to the high economic activity in this sector. Likewise, The Food and Agriculture Organization (FAO) estimates that 6% of global food losses occur in this region, about 15% of the available food [43]. According to the latest renewable energy inventory carried out in Colombia, around 72 million tons of residual agricultural biomass are produced each year [44]. This amount results from processing products such as coffee, oil palm, rice, sugar cane, banana, and plantain [45]. Coffee is one of the most important products worldwide, around Latin American, Asian, and African countries have coffee crops. Colombia is the second coffee producer, and this product is the most relevant, with a sown area of 844.744 ha (2020), and a production of 66 tons in October 2021 [46]. In coffee-derived products, 60 wt.% of the fruit is used, and 40 wt.% is considered waste (leaves, husk, stem) [47]. As well, it is estimated that sugarcane is responsible for the production of 6.5 million tons of residues per year [48], rice sector 700 thousand tons of rice husk [49,50], and palm oil crop residues are roughly 1.6 million tons per year [51], and so on for other products. 

In recent years the efficient transformation of these residues has gained interest in reducing environmental pollution, minimizing the use of natural renewable and no renewable resources, and generating employment and economic resources. The use of solid waste is an alternative that promotes technological development oriented towards a sustainable transformation of natural resources [52].

### 2.3. Waste Valorization

**Figure 5:**
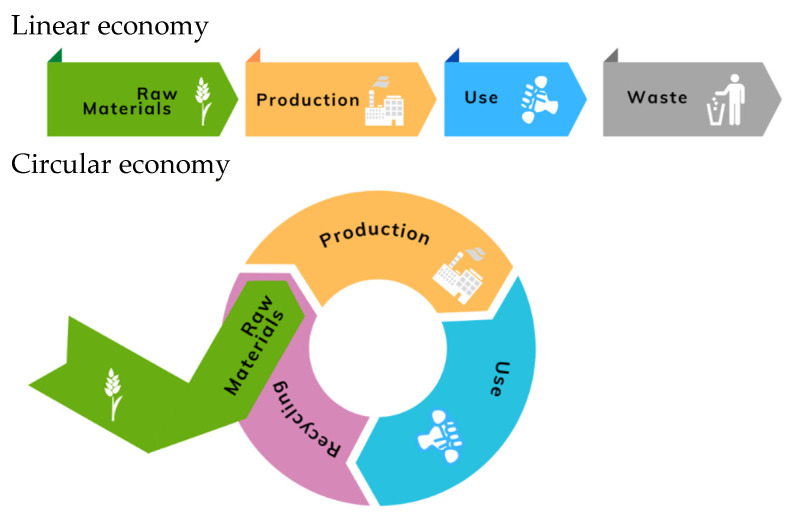
Linear and circular economy models.

The above challenges present an excellent opportunity for developing a CE using new technology and creating profitable businesses to address the utilization of plastic and agro-industrial wastes. For many years, linear extract-produce-use dump materials and energy flow have been followed [53]. The main characteristic of this economic concept is that the waste resulting from the production processes is discarded into the environment, which assumes boundlessness and easy availability of material resources (Figure 5).

Unsustainable resource consumption, product redundancy, waste, and pollution must be avoided. For this reason, design for a CE has recently become a research area in sustainable design [54], intending to keep materials in use through as many life cycles as possible. The core of the CE is the circular flow of materials and the use of resources through multiple phases [55], resulting in a benefit to the society and the economy as a whole by reducing the natural environment’s usage as a sink for waste and reducing the use of virgin materials for economic activities [56]. Embrace of CE model would reduce the need to manufacture new materials and remove the generation of waste products using them in high-value applications. 

Concerning plastic waste, the development of a circular economy offers the opportunity to deal with environmental pollution issues and encourage global material flow with high resource efficiency to create an effective after-use plastics economy. An important pathway to keep plastics within the system for as long as possible is recycling, which also can bring opportunities for innovation, competitiveness, and new jobs. According to the British Plastic Federation, recycling can create 25,000 additional jobs in the industry in the UK by 2030 [57]. However, nowadays, recycling rates are low, with 4% just in the plastic packaging field [58], and in Colombia, the recycling rate is low [59]. However, foundations such as Ellen MacArthur promote initiatives to enhance the plastic circular cycle as the ‘The New Plastics Economy’. This plan aims to create an effective after-use plastics economy, reduce leakage of plastics in natural systems and decouple plastics from fossil feedstocks [60].

Another potential alternative to minimize waste issues is exploring the role of renewable resources. Over the last decades, several studies have evaluated agro-industrial wastes as new materials in different production chains [61], such as chemicals, materials, and fuels. Most agro-industrial waste is lignocellulosic biomass (hemicellulose, cellulose, and lignin), recognized for its mechanical and thermal properties, availability, low cost, and biodegradability [62]. 

### 2.4. Colombian Perspective

CE adoption is also driven by the 2030 Agenda and the Sustainable Development Goals (SDGs) to minimize climate change, close social gaps, and conserve natural wealth, among other aspects supporting the reduction of the ecological footprint of human activity [63]. To achieve the objectives of this Agenda, in Colombia in 2018, the Government presented the “National Strategy of Circular Economy”. This strategy aims to increase the rate of recycling and waste utilization, which today stands at 8.7%, so that it rises in the year 2030 to 17.9%. Also, it promotes innovation and value generation of production and consumption systems through optimization, change, recycling, and generating materials, water, and energy. Further, it stimulates the development and implementation of new business models between companies, consumers, and stakeholders [64].

Besides, the Mision Internacional de Sabios is a group of national and international experts whose objective is to contribute to the construction and implementation of public policy on education, science, technology, and innovation focused on the Colombia Sustainable and Productive Challenge. This group of experts identified eight principal areas where converging technologies, biotechnology, environment, and bioeconomy can be highlighted. They also identified the importance of the use of technology and 4.0 Industry to the country’s transformation through a model of sustainable bioeconomy and creative economy industries and services with high technological content and CE companies that take advantage of the renewable resources [22]. 

Colombian biodiversity represents a high potential in the manufacturing industry due to its ability to transform into green materials, which can be applied in many industrial fields, from energy to health. However, technological development is also necessary to achieve effective resource utilization.

## 3. New Technologies: 3D Printing

Developing new and more sustainable technologies such as artificial intelligence, robotics, additive manufacturing, neurotechnology, biotechnologies, virtual and augmented reality, new materials, and energy technologies have also been driven by industry 4.0. The emergence of these technologies can create opportunities to change manufacturing distribution and the flow of materials and goods with many potential sustainability benefits. They allow the creation of manufactured products to minimize adverse environmental impacts and conserve natural and energy resources, representing a potential to move towards the CE [65,66].

One of these emerging technologies is additive manufacturing (AM). The use of AM in developing products, spanning conceptual design, functional parts, and tools, has been increasing in the last few years due to its versatility and simplicity [67]. With AM, the user can create parts with complex geometries from a digital representation without a mold or an extraction operation, as the traditional manufacturing techniques such as injection molding, machining, or die-casting, provide shorter distances for distribution, and allow the production of customized parts [19,67]. This technology is considered to be promising for sustainable manufacturing because its digital and additive nature provides the opportunity to save raw materials, increase product functionality, reduce energy consumption, and also enables on-demand production of spare parts for repair when compared to subtractive technologies [67,68,69]. The role of AM in the 4.0 industry is not about replacing mass production; it is to manufacture components that cannot be produced by conventional methods [70]. This manufacturing technique allows the production of personalized items while maintaining low prices; it does not need the additional fees of the molds and tools for customized products required in traditional manufacturing techniques [71].

An AM process that has increased its use in the last decades is the 3D printing technology, which involves objects manufactured in specific layers. The global 3D printing market in 2021 was USD 12.6 billion [72], and it is estimated that the printer segment represents the largest share of the 3D printing market. 3D printers can be used for personal, professional, and production purposes. Some of the most used techniques are fused deposition modeling (FDM), laminated object manufacturing (LOM), stereolithography (SLA), and selective laser sintering (SLS), among others. Techniques are selected based on the material used and the applications needed.

**Figure 6:**
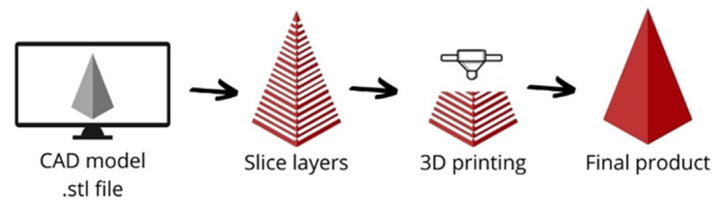
3D printing process diagram.

Although there are many 3D printing techniques, they all follow the same principles. First, the printable model is designed using a computer-aided design program (CAD). Then, the model’s geometry is defined by its conversion to a standard triangle language (STL). The file is processed in a slicer software, where the printing parameters are defined, and the model is cut, producing a path followed by the print head. A code is obtained due to the model processing in the slicer software. The code instructs the printer to create the physical object; sometimes, an end-part finishing is required [73]. Figure 6 presents the 3D printing process flow diagram. 

### 3.1. Fused Deposition Modeling (FDM)

**Figure 7:**
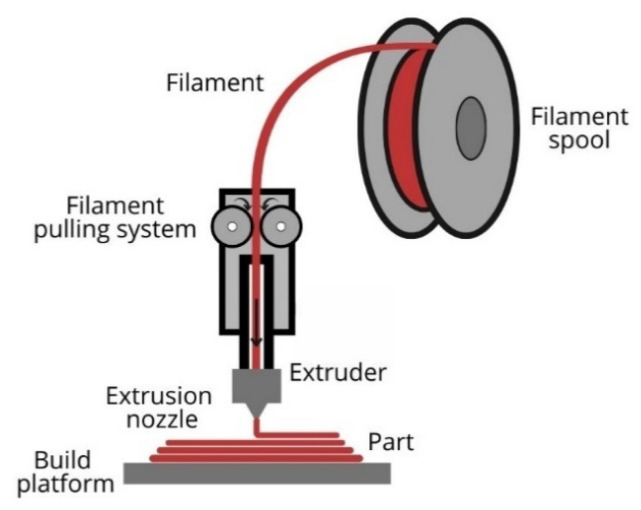
FDM mechanism.

FDM, also known as fused filament fabrication, is the most used 3D printing technology for polymers due to its simplicity of operation and the high availability of printers [74]. During this process, parts are built using thermoplastic material in a filament shape. The FDM process’s basic principle is to melt the raw material and use it to build new shapes. The filament is unwound from the spool and fed into a liquefier, by two drive wheels, where the material is heated until its melting temperature. Then, the semi-liquefied material is extruded through a nozzle that moves according to the set code and deposits it layer-by-layer, following the determined path, on the heat-built platform of the printer. During the process, the solid filament works as a piston to push molten material through the nozzle. The deposited filament fuses with the previous one, then cool down and solidify. The bed temperature is maintained at a lower temperature to make easier the solidification process (Figure 7) [75,76]. To produce a part, almost all the feed material is used; therefore, less material is wasted for each print [19]. Dual nozzle printers are also available, where one is used to build the part and the second dispense the material for the support structure, or both are used in case compositional gradients are required [75]. The 3D printed parts’ resolution and quality depend on the material’s properties and the 3D printing parameters. Printing parameters are specified in the slicing software; it includes printing speed, raster angle, melt flow rate through the nozzle, layer thickness, infill density, build orientation, extruder temperature, and bed temperature, among others [75]. 

The Fused Deposition Modeling technique applications can be seen in many fields, from producing prototypes and small parts to large components in vehicles or airplanes. The top five manufacturing industries where FDM is used are automotive (32%), customer products (18%), business machines (11%), medical (9%), and education (9%).

Table 1 summarizes some of the potential applications of FDM technology. FDM process is frequently used in automotive sectors for prototypes and end-user products. Polymers such as polycarbonate, nylon, or polyetherimide are used to obtain parts with desired mechanical properties and good dimensional accuracy. In medical applications, FDM supports unique requirements like the ease of access, high complexity, small production quantities, patient-specific needs, and customization [77].

In Latin America, interest in the use of additive manufacturing still growing [79]. The 3D printing market in the region is about 5% of the world. The region’s most common applications of 3D printing are in education, followed by service providers (prototypes) and medicine. Currently, Mexico has more desktop and professional 3D printing brands, followed by Brazil and Colombia. The arrival of companies such as HP, 3D Systems, Big Rep, and Ultimaker is worthy to note. Also, there are local 3D printing machine producers such as Colibrí 3D in Mexico or Fused Form in Colombia [80].

According to the Observatorio de Economía Digital, in Colombia, only 3% of the companies used 3D in 2018, and 4% have plans for its implementation. Also, 4.8% of big companies have implemented 3D printing technology [21]. In Colombia, experiences such as Fabrilab, Conconcreto, Undos3D, and Fused Form are known. Fabrilab, which started in 2015, manufactures low-cost customized prostheses for children to ensure a good fit physically and emotionally [81,82]. From the industry, Conconcreto developed the first 3D printer of concrete with Siemens technology to produce prefabricated parts, such as intern walls, facades, houses, and urban furniture, reducing the time and costs of construction [83].

Moreover, Undos3D gives medical solutions such as 3D printing of bio models based on tomographs to have previsualization before surgery and anatomic modeling [84]. Other companies, such as MakerR and Fused Form, are pioneers in 3D printers’ production. Fused Form has introduced a new large-format pellet extrusion 3D printing system, the first in the country, allowing lower material costs and expanding the variety of plastics that can be printed [85]. In the last years, searching for a quick implementation solution to face COVID-19 and support doctors, different universities and entities have developed and manufactured anti-fluid masks, respirators, valves, and other accessories [86,87]. 

### 3.2. FDM Feedstock Materials

FDM printing also allows various materials, from traditional polymers to bio-composites containing natural fibers, such as wood or cork [88]. Nevertheless, there are some properties that materials for FDM requires, such as low melting point and reduced viscosity, to flow through the nozzle for deposition and adhesion to the previous layer [88]. Thermoplastic polymers are the most used materials in fused deposition modeling techniques due to their high processibility, low cost, low moisture absorption, and easy manipulation [89]. Amorphous thermoplastics are preferred because they present low shrinkage levels, which is primordial to the dimensional accuracy of the final product. Of the different thermoplastic filaments, the most common are poly-lactic acid (PLA) and acrylonitrile butadiene styrene (ABS). PLA filament is derived from renewable resources by the polymerization of sugars and starches and is known for its ability to biodegrade but has poor mechanical properties [90,91]. ABS is derived from processing petroleum and exhibits excellent mechanical properties; however, it produces an unpleasant odor during processing [91]. Researchers from the United States Environmental Protection Agency (EPA) found that both PLA and ABS produce particles of an ultrafine size that represent a potential for exposure to respirable particles [92]. Other commonly used materials are polypropylene (PP), thermoplastic polyurethane (TPU), polyethylene (PE), Nylon, and polycarbonate (PC). Overall, polymers with melting temperatures below 300 °C are suitable for 3D printing because the 3D printing temperature is around 250 °C [93] (Table 2). However, nowadays, it is possible to print materials such as polyether ether ketone (PEEK), whose melting point is around 360 °C [94]. 

### 3.3. Fused Deposition Modeling (FDM) and Circular Economy (CE)

Although FDM has many advantages, it also presents some challenges to environmental sustainability [98]. These issues are, for example, the use of raw materials, the waste generated, the overexploitation of the technology, the release of volatile organic compounds, and the energy consumption used during the production of a part [99]. Gebler et al. [100] identified different criteria to evaluate 3D printing sustainability, such as economy, production costs, environment, process energy, process emissions, lifecycle, and recyclable waste. Recently, an important number of studies related to the Life Cycle Assessment (LCA) of AM technologies have been done with the principal aim of providing a framework to improve the sustainability of AM processes. For example, Paris et al. [101] compared the LCA between additive and subtractive techniques. The study focused on selecting an optimal manufacturing strategy for an aeronautical turbine following the ISO 14044 standard. Obtained results demonstrate that AM technologies require lowers energy consumption, implying less CO_2_ emissions and lower economic costs. Camposeco et al. [102] evaluated the optimization of 3D printing parameters intending to reduce 3D printing time and, with it, also minimize energy consumption, as electricity is obtained from fossil fuels. In addition, Gebler et al. [100] demonstrate the sustainable energy impacts of total primary energy supplies (TPES), which present a decrease during the entire product lifecycle. 

**Figure 8:**
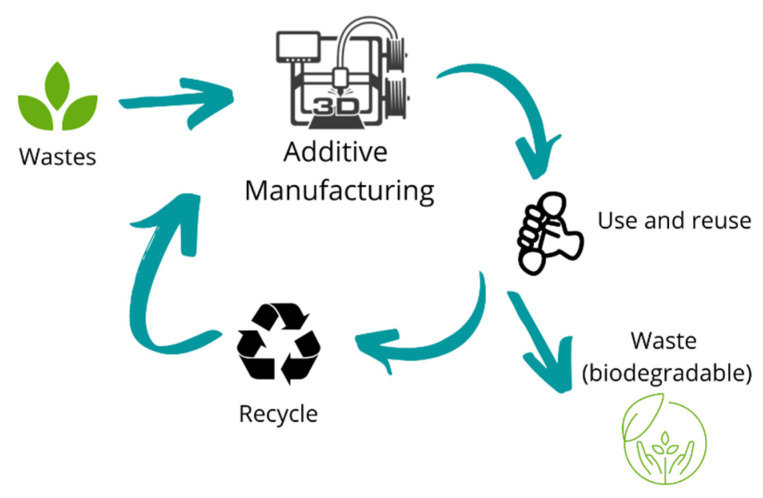
Additive manufacturing in a circular economy model.

Another critical part of the environmental impact of FDM is related to the materials used as raw materials. Most of these materials are petrochemical-based materials that are hard to degrade if discarded into the environment at the end of their life cycle. In addition, the large amount of wasted plastic demonstrates a linear model of production and consumption. The CE represents a potential alternative to this linear model by keeping in use materials as many life cycles as possible, reducing the need to produce new plastic and plastic waste generation. At the same time, budgets and the environment benefit. In this way, changing petroleum-based material to more sustainable renewable, non-toxic, compostable, recyclable, and abundant is an opportunity to make FDM a more sustainable manufacturing technique [103]. Also, sustainability for 3D printing materials is important since 3D printing is projected to grow 15.6% between 2020 and 2025 [104]. Figure 8 represents the lifecycle of AM products based on the circular economy CE concepts.

The CE in FDM, and additive manufacturing in general, allows the use of local materials, promoting in-situ recycling and upcycling, which could lead to transportation, and packing reduction, thus the environmental pollution caused by air and plastic pollution [105,106]. A fully circular economy seeks to use and reuse existing materials, adding value to post-consumer products and returning them to the production chain [107]. Therefore, recycling is one of the best solutions to treat post-consumer plastics following the circular economy principles [108]. The industry is implementing recycled plastics (ABS, PLA, PET) as raw materials to develop filaments for 3D printing applications. In the case of PLA, although it degrades over many use cycles, it is still an attractive material due to its renewable planted-based source nature. As an example, companies such as Reflow, Formfutura, GreenGate 3D, Kimya, Filamentive, Re-pet 3D, Nefilatek, and RePlay 3D, among others, have been specializing in the production and commercialization of recycled-based filaments for FDM, using from 25 to 100% of recycled PLA, ABS, high-density polyethylene (HIPS), PETG, acrylonitrile styrene acrylate (ASA), and PC [109,110,111,112]. Others, like 3devo, are developing machinery to process post-consumer waste and convert it into quality 3D printing filament [113]. 

Recycled plastic has been studied and implemented as raw materials in 3D printing, but agro-industrial waste has gained recognition in this field by encouraging the utilization of biobased materials that can be recycled and reused. Companies from Italy (Canapuglia), Netherlands (FormFutura), Ireland (3D-Fuel), and Poland (Pri-Mat3D) are innovating with filaments based on PLA, filled with agro-industrial waste from industries such as tomato, pomegranate, coffee, and beer, and others filled with natural fibers as hemp, wood, bamboo, or cork [114,115,116].

## 4. Sustainable Materials for 3D Printing: Giving Value to Waste

Researchers are also developing new materials for this manufacturing technique to support the industrial conversion to a more sustainable production chain, allowing the decrease in global plastic usage and considering recycling as a viable strategy for reducing plastic waste [117]. Genuine sustainable materials can be achieved by considering the whole lifecycle, from the origin of the raw material to its waste management. Therefore, polymers’ and composites’ sustainability are not only about the choice of renewable or environmental-friendly resources for their production, but also about minimizing their environmental impact.

### 4.1. Polymeric Recycled Matrices

**Figure 9:**
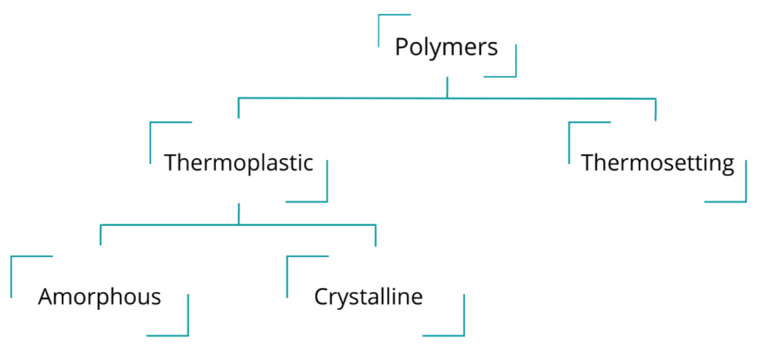
Plastic classification.

Environmental pollution associated with plastic waste production is a significant concern. Recycling represents a potential solution to plastic restrictions and environmental awareness. Nonetheless, not all types of plastics can be recycled. One way to classify polymers is by their mechanical and thermal behavior. Figure 9 presents the major polymer categories. Thermoplastic polymers are composed of long chains joined through relatively weak van der Waals bonds. They typically behave in a plastic, ductile manner, and upon heating, they soften and melt, allowing their processing to elevate temperatures [118]. Some examples of thermoplastic polymers are polyethylene, nylon, PET, PP, and PVC. 

On the other hand, thermosetting polymers are composed of long chains joined by cross-linked solid bonds. The behavior of thermosetting polymers tends to be stronger but more brittle than thermoplastics. In contrast, they do not melt upon heating, for they cannot easily be reprocessed, so recycling is difficult [118]. Phenolics, amines, polyesters, epoxies, urethanes, and silicones are some examples of thermosetting polymers.

According to the Society of the Plastics Industry, recyclable thermoplastics are classified into seven types. (1) Polyethylene terephthalate (PET), used in packaging, fiber clothing, automotive parts, and biomedical applications, is the most recycled plastic globally. (2) High-density polyethylene (HDPE) is one of the easiest plastic polymers to recycle. (3) Polyvinyl chloride (PVC) is not recyclable in normal conditions. (4) Low-density polyethylene (LDPE) is a low-quality plastic for which its recycling is not feasible from a financial point of view. (5) Polypropylene (PP) can be recycled. (6) Polystyrene (PS) is not recyclable in normal conditions. (7) The last category is “Others”, which includes bioplastics, composite plastics, plastic wrapping paper, and polycarbonate (PC), which not all of them can be recycled [119].

Even though about 90% of plastic can be reused globally, just 9% is adequately recycled [120]. The transition towards a circular economy can be achieved with changes in waste management and different value chain steps, like design and production. This creates a need to study how to design, produce, use and recycle plastic within the circular economy [121]. The end-of-life phase involves the collecting, recycling, and lifecycle assessment [122]. Distributed recycling of plastic waste for additive manufacturing is one recycling method that involves economic advantages [107,123]. Plastic recycling is done through chemical or mechanical processes. During chemical recycling, the polymer is wholly or partly depolymerized through a chemical reaction allowing the production of a similar or the original plastic. However, this mechanism is usually necessary for a catalyst, making this option economically inefficient due to the catalyst’s cost and the product’s narrow fractional composition [124]. Otherwise, mechanical recycling is used just for processing thermoplastic polymers, where they are ground down, melted, and compounded to produce a new component, such as feedstock filament for 3D printing [124]. Nowadays, extruders to transform polymers in 3D printing filaments have been developed and are commercially available, including Filafabot, Noztek, Filabot, Filastruder, and Filamaker, among others [125]. 

**Figure 10:**
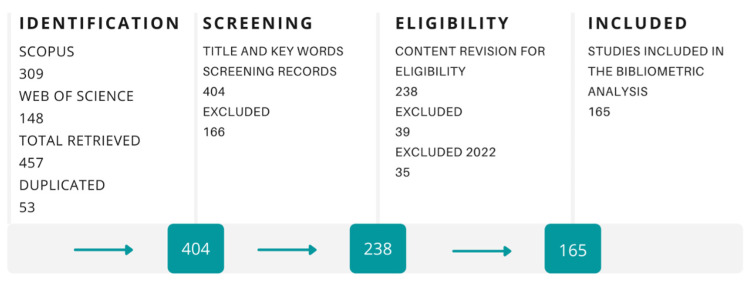
Literature review methodology for recycled polymers used in FDM.

**Figure 11:**
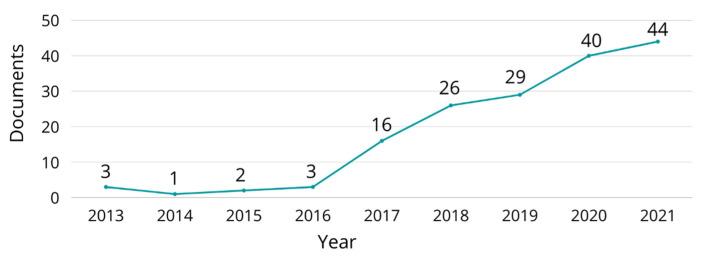
Temporal distribution of the studies where recycled plastics was evaluated as possible feedstock in FDM until 2021.

Further, scientists and academics are developing techniques to produce 3D printing filaments based on recycled polymers. An advantage in this field is that, in general, polymers that can be melted without degradation are potential candidates to be used in the FDM technique. Afterward, developed materials must undergo rheological, thermal, and mechanical characterization to evaluate the feasibility of using a particular material. Tendencies in 3D printing of recycled polymers through FDM were identified by bibliometric analysis. The bibliometric analysis was done following the Systematic Review Guide proposed by Siddaway et al. [126]. Scopus and Web of Science were the databases for the literature research and the detailed process is described in Figure 10. Also, Figure 11 shows a temporal distribution of studies that developed 3D printing filaments for FDM applications based on recycled polymers. In the last years, recycled polymers have gained interest due to the importance of environmental concerns and new policies that different governments have implemented to minimize the environmental impact caused by plastic pollution.

Different studies have been performed to evaluate the feasibility of recycling plastics. Zander et al. [127] focused their investigation on developing and characterizing 3D printing filaments from PET bottles and packaging without modifications or additives with tensile strength (35.1 ± 8 MPa) comparable to commercial polycarbonate-ABS filaments. Also, the three-point bending tests showed similar properties between recyclable PET and commercial PET printed parts. Results demonstrated that recycled PET filaments could replace commercial filaments. Vaucher et al. [128] evaluate the possibility to develop PET filaments form beverage bottle waste and the potential addition of HDPE from bottle caps and rings. Results showed that the HDPE presence does not affect the extrusion process or the 3D printing quality of the filaments. However, the HDPE addition contributes on the materials’ toughening. Oussai et al. [129] compared the mechanical properties of 3D printed parts made of recycled and virgin PET. Recycled PET increased by 14.7% and 2.8% for tensile and shear strength, respectively. This result is due to microscopic changes that could occur when layers are interlocking during their deposition. Nevertheless, there was no difference in tensile modulus. They finally concluded that the demand for improved recycled 3D printing filament technologies is heightened due to the comparable properties of both evaluated materials. Pepi et al. [130] developed recycled PS, PET, and PP filaments and printed them into tensile bars. PS tensile properties were brittle, with a mean tensile strength of 19.9 MPa, about half that of commercial PS filament (34.0 MPa). PET filament obtained from plastic bottles showed a comparable tensile strength with the commercial PC-ABS filament, with 36.4 and 34.8 MPa, respectively. The PP exhibited a lower mean tensile strength with 20.1 MPa. Then, Zander et al. [131] presented the development and characterization of recycled polymeric blends, PP/PET, and PP/PS. Polymer blends are attractive for reusing waste streams at a lower cost, but mechanical properties could not be improved compared to those with only one polymer. Nevertheless, PP is immiscible with PS and PET, and additives such as styrene ethylene butylene styrene (SEBS), and grafted PP are used to improve the interaction between polymers.

According to the mentioned studies, it is possible to use recycled polymers for 3D printing applications. Nevertheless, mechanical properties are not the only point evaluated in this kind of materials. Kreiger et al. [105] and Baecher et al. [109] investigated the life cycle of recycled HDPE as a 3D printing feedstock. Regarding energy consumption, Baecher et al. [109] found that, energetically, homemade HDPE filaments can be 40 times more economical than purchasing filaments commercially. In addition, some challenges of using recycled polymers have been identified. Cruz et al. [23] observed different recycling cycles’ influence on PLA 3D printing feedstock. Results showed the feasibility of using recycled PLA in 3D printing, mechanical properties of 3D printed parts for the first cycle support that recycled PLA can be comparable in tensile strength and elastic modulus to the parts printed with commercial PLA. Nevertheless, mechanical properties tend to decrease when many recycled cycles are implemented. 

In Colombia, the development of FDM feedstock based on recycled polymers has been limited. Some universities are focused their research on this field. For example, Pulecio, from the Fundación Universitaria Empresarial de la Camara de Comercio, presented an educational proposal to recycle PET bottles and implement them in developing 3D printing filament [132]. In addition, at Universidad Autonoma de Occidente, the development of 3D printing filaments based on recycled polymers has been investigated. Studied plastics were PET, PP, HDPE, and PS. HDPE presented the best behavior during filament manufacturing and a homogenous diameter. Even though PP presented a fluid processing behavior during extrusion, the resulting diameter varies, representing an inconvenience for the continued 3D printing. PS has good behavior inside the extruder; however, it turns fragile when the material comes into contact with room temperature [133].

### 4.2. Natural Fiber Composites

Biocomposites are other commonly used materials that have gained importance in the scientific field. Biocomposites have also gained significant traction in the market, with a forecasted value of US$7.8 billion by 2024 [134]. These materials are composed of at least one natural resource, allowing the development of materials with matrixes, ranging from biopolymers to petroleum-based virgin or recycled polymers [135,136], filled with natural fibers. 

Natural fibers could be obtained as a powder or chopped depending on the resources. Powder particles, such as wood, are obtained as waste from their processing, such as planer shaving or molding. On the other hand, bast fibers are obtained from agricultural waste, like jute, kenaf, bamboo, hemp, or rice husk. Some properties that make natural fibers attractive to be implemented in the industry are their low cost, low abrasion, low density, high availability, and biodegradability, making natural fibers a potential alternative to replace synthetic ones [137]. 

**Figure 12:**
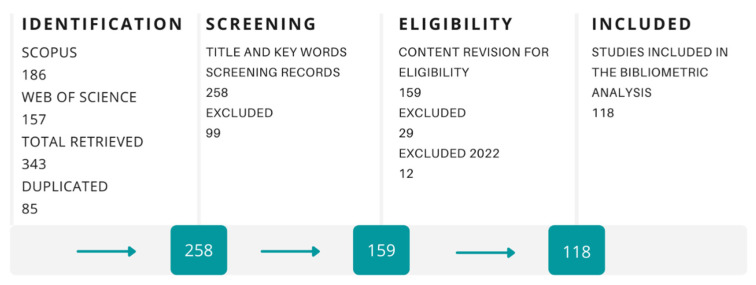
Literature review mechanism for natural fibers used in FDM.

Regarding the sustainability advantages of biocomposites, if their manufacturing is performed with local resources, it will contribute to sustainable regional development. Also, the longer the product life of natural fiber-based biocomposites, the more significant the environmental benefits [138]. Further, its applications can contribute to sustainability. For example, the lower weight of natural fibers makes them suitable for producing automotive parts, reducing fuel consumption and gas emissions [139]. Other common application includes building materials, such as architecture moldings, decking, and railing components [140]. Thermoplastics are promising matrices to use with natural fibers due to their low cost, high availability, and low processing temperatures, allowing for material processing while avoiding natural fibers’ thermal degradation [141]. To identify tendencies in using natural fiber biocomposites in FDM, a bibliometric analysis was done following the literature review mechanism proposed by Siddaway et al. [126]. The detailed process is shown in Figure 12.

**Figure 13:**
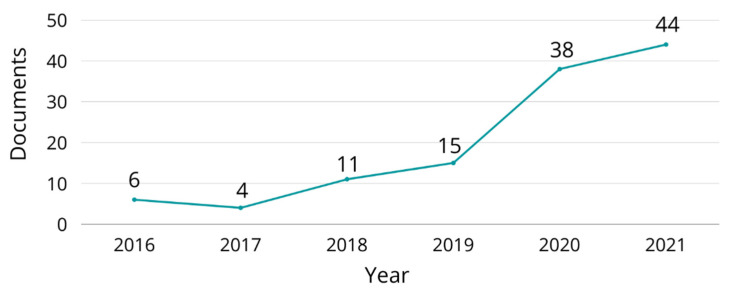
Temporal distribution of the studies where natural fibers was evaluated as possible feedstock in FDM until 2021.

According to the reviewed literature, the temporal distribution of studies where natural fiber composites was performed is shown in Figure 13. The popularity of natural fiber biocomposites in FDM has increased significantly due to their sustainability, low density, and thermal and noise insulation properties. Wood flour is one of the most studied natural fibers in FDM applications. Small diameter and unutilized wood particles cause wildfires [142]. Tao et al. [143] presented the development and characterization of net PLA and wood flour (5 wt.%) filled PLA 3D printing filaments to determine the effects of the filler on some properties. They obtained a composite filament suitable to be printed. Through SEM analysis, they concluded that the fiber changes the microstructure of the PLA fracture surface. Tao also identified the poor interfacial adhesion between the wood flour and the PLA matrix and justified this behavior because the wood is hydrophilic and has a polar surface, while the PLA is hydrophobic and has a non-polar surface. Regarding mechanical properties, they obtained a lower strength for the composites due to the broken wood/PLA interface. Kariz et al. [144] evaluated the effect of wood content-filled PLA 3D printing filaments on 3D printed parts. They evaluated 0, 10, 20, 30, 40 and 50 wt.% wood contents. The density of the 3D printed parts decreased with increasing fiber content. Although with 10 wt.% of wood, the 3D printed parts showed an increase in tensile strength from 55 MPa to 57 MPa, with higher fiber content, the strength decreased, obtaining a tensile strength of 30 MPa when 50 wt.% of fiber was used. The fiber had a rougher surface on the 3D printed specimens’ surface, with voids and visible gaps between the wood and PLA. Kain et al. [145] not only evaluated the fiber content in composite filaments (15 and 25 wt.%) but also evaluated the infill orientation (0°, 15° crossed, 30° crossed, 45° crossed, 60° crossed, 75° crossed, and 90°) effect on 3D printed specimens. Concerning the infill orientation, they concluded that there is a direct interaction between infill orientation and mechanical performance. In addition, they observed that a higher fraction of wood resulted in better mechanical properties. Petinakis et al. [146] studied the interfacial adhesion between wood flour and PLA using methylene diphenyl diisocyanate as a compatibilizer. They obtained similar results as other studies. The composites presented poor interfacial adhesion represented by visible voids, particle pullout, and fiber/matrix interface gaps. Also, a decrease in break elongation and an increase in the modulus due to the rigidity of wood particles were observed.

Table 3 presents other natural fibers filled with biocomposites in FDM. Le Duigou et al., Depuydt et al., and Zhang et al. are some authors studying the implementation of continuous flax fibers in 3D printing filaments. Le Duigou et al. [147] obtained flax/PLA biocomposite filaments that exhibited irregularities on their surface and low fiber dispersion in the PLA matrix. However, obtained mechanical properties increase compared to short fiber-filled PLA. Depuydt et al. [148] developed a prepreg method to manufacture 3D printing filaments. They increased tensile and flexural properties compared to the PLA 3D printed specimens. The tensile strength and modulus increase by 89% and 73%, respectively. Flexural strength and modulus improve by 211% and 224%, respectively. Other continuous fibers have been evaluated, such as the jute. Marsuzaki et al. [149] compared jute/PLA, continuous carbon fibers/PLA, and neat PLA 3D printed composite specimens’ mechanical properties. The main conclusion of this study was that even though carbon fiber composites have better mechanical performance than jute composites, the latter has better mechanical resistance than the neat PLA specimens. Hemp fiber was evaluated as PLA reinforcement by Yacuchi et al. [150] According to the obtained results, hemp/PLA composites exhibited an increase in tensile strength from 46.8 MPa to 65.3 MPa compared to the neat PLA matrix. Meanwhile, They identified that the composite specimens deteriorate faster than the PLA.

Particulate fiber such as bamboo, rice husk, rice straw, and cork has also been investigated. Osman et al. [158] studied the influence of different rice straw content (0, 5, 10, 15 wt.%) in 3D composite ABS-based filaments in the mechanical properties of 3D printed specimens. Tensile and flexural properties (strength and modulus) decreased as the rice straw content increased; however, at 15 wt.%, the fiber flexural properties increased. The 3D printing parts exhibited more porosity as the fiber content increased, which explains the mechanical behavior. As obtained with continuous fibers, SEM analysis presents spaces between fiber and ABS matrix due to the poor bond. Girdis et al. [159] studied Macadamia nutshell as ABS filler. They obtained a reduction of more than 27.4% in density with the addition of 29 wt.% of fiber, compared to the neat ABS. Concerning tensile properties, tensile strength decreases when fiber content increase, but tensile modulus increases. Natural fibers, specifically those with high lignocellulosic content, reduce the crystallization of the polymeric matrices allowing easy printability. Nguyen et al. [161] obtained an easy printable nylon filament thanks to the lignin addition. Lignin fibers retards nylon crystallization, leading to low-melting imperfect crystals. Also, Morales et al. [162] obtained a reduction in the warping effect in recycled polypropylene 3D printed parts when rice husk is added. According to the analysis, natural fiber composites have the potential to be manufactured through different manufacturing techniques obtaining competitive properties for applications where synthetic materials are used. Natural fiber composites could lead to the circular economy transition, adding value to agro-industrial residues while minimizing the waste generation of agricultural processes.

### 4.3. Natural Fiber and Recycled Matrix Biocomposites

Furthermore, the opportunity to use recycled polymers filled with agro-industrial waste or natural fibers results in the production of lower environmental impact products. Waste stream volumes can be minimized while their second-life applications maintain the material value. Among the fibers that have been evaluated, in recycled polymer matrixes fillers, are the harakeke, hemp [163,164], banana [165], and wood [166]. 

Stoof et al. [163] evaluated different harakeke and hemp fibers (0–50 wt.%) as pre-consumer recycled polypropylene. Maleated polypropylene and an alkali treatment with sodium hydroxide were used to produce 3D printing composite filaments. According to previous studies, different alkali treatment parameters were implemented for each type of fiber. Filaments with more than 30 wt.% of fiber exhibited no dimensional consistency to be applied in 3D printed technology, so those 3D printing filaments were discarded from the research. Tensile strength and Young’s modulus increase with fiber content and improve considerably compared to the pre-consumer recycled polypropylene. This research studied the warping effect of 3D printed specimens based on different configurations. The most significant improvement was obtained using 30 wt.% of harakeke fiber (0.34% of warping) compared to the neat recycled polypropylene (84% of warping). Pickering et al. [164] also studied adding different fiber content of harakeke and hemp alkali treated fibers but using post-consumer polypropylene based on plastic bags. Obtained results were similar to those obtained by Stoof et al. Tensile strength and Young’s modulus increase with increasing the fiber content. They also highlight the importance of well-dried filaments before 3D printing. Undried filaments have greater moisture and voids content, reducing mechanical performance. They showed that fiber is an effective additive for reducing shrinkage. Singh et al. [165] evaluate the possibility of developing 3D printing filaments based on recycled ABS filled with banana fiber (5 wt.%). The banana improved the load carrying capacity of ABS: by 16% and 11% in the peak load and break load, respectively. However, the elongation and Young’s modulus decreased by 13% and 52%, respectively.

Recycled polymers filled with agro-industrial waste or natural fibers are a field that has not been widely studied. This represents an interesting field to focus on, trying to reuse plastics and give them a longer life cycle.

### 4.4. Natural Fiber Biocomposites: Colombian Perspective

In Colombia, two of the principal economic activities are agriculture and agroindustry. Different by-products and residues are produced during agricultural product processing. Industry and academic fields have been interested in finding ways to manage these residues and give them added value. One explored field to apply them to is composite materials. Some of the studies developed in Colombia focus on developing new materials based on Colombian natural fibers through different manufacturing techniques. Wasted biomass results from industries such as rice [167], chambira [168], fique [169,170,171,172], coffee [173], sugarcane [174], cotton [175], banana [176], plantain [177], pineapple [176], sisal [178], hemp [178], and Manicaria saccifera [179,180] has been studied. Molding (injection and compression), hand lay-up, and vacuum infusion are the most used manufacturing techniques.

3D printing is a technology that, in Colombia until now, is only beginning to be implemented by some companies. Consequently, developing new and more sustainable materials for this technique has not been extensively explored. Montalvo and Hidalgo studied sugarcane bagasse reinforcement in 3D printing filaments using different thermoplastic matrices, PE, PP, PLA, and ABS. The mixture between PP and 20 wt.% of sugarcane presented the best behavior in the extrusion process. Therefore, 3D printing, and mechanical performance were evaluated with this configuration. According to their study, natural fiber inclusion act as a filler rather than a reinforcement, but they demonstrate the viability of 3D printing with local sub-products, such as the sugarcane bagasse. To improve the mechanical and rheological behavior of the materials, they suggest treating the fibers before the composite manufacturing and implementing different additives to improve the interaction between the fiber and the matrix [181]. Cerpa et al. [179] evaluate the mechanical performance of a sustainable material based on Manicaria Saccifera palm waste and PLA. The fiber weight ratio, fiber treatment, and 3D printing raster angle were evaluated. Accordingly, lower mechanical properties were obtained with the addition of fiber. However, with the chemical treatment of the fiber, mechanical properties were on par with the material with fibers without treatment. 

**Figure 14:**
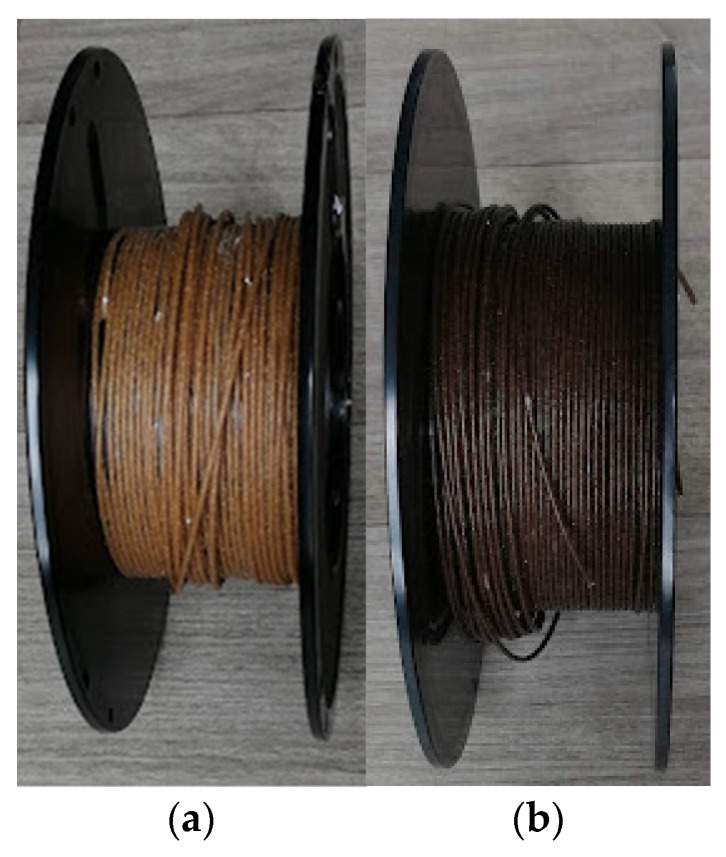
3D printing filaments based on Colombian agro-industrial waste, (**a**) recycled PP/rice husk 10 wt.%, (**b**) recycled PP/cocoa bean shell 5 wt.%.

3D printing filaments manufactured with recycled polymers and Colombian natural fiber are a relatively unexplored field. Morales et al. [162,182] evaluated the influence of adding natural fibers, like rice husk and cocoa bean shell, on 3D printing filaments based on recycled polypropylene on 3D printed parts performance. Developed materials are shown in Figure 14. Results showed that particulate fiber acts as a filler due to the poor interaction between the filler and the matrix. This behavior was observed by SEM analysis, where gaps between both components are visible. Strategies such as treating the fiber or implementing additives are also suggested. 

### 4.5. 3D Printing Materials’ Development Challenges

**Figure 15:**
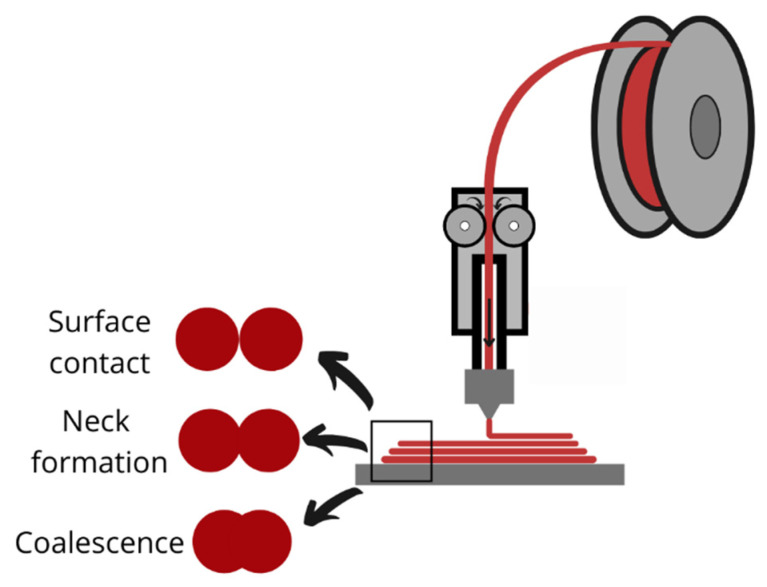
Layer interfacial bonding mechanism.

Different challenges have been identified regarding the FDM 3D printing technique from a technological perspective. The layering process adds stress concentration points and voids, causing a reduction in 3D printing parts quality and mechanical properties compared to other manufacturing techniques [183]. The part’s integrity depends on the bonding phenomena and quality between adjacent filaments in a layer and successive layers. Gurrala et al. [184] and Aida et al. [185] observed that differences between layers’ temperatures are insufficient to have a fully coalesced neck (Figure 15). FDM technique has an anisotropic nature; consequently, parameters such as the building plate’s fabrication orientation and the printing rater angle can determine 3D printing parts’ mechanical properties [186]. In addition, other printing parameters affect the mechanical properties, such as the nozzle diameter, temperature, and infill density. 

Nowadays, the largest polymer 3D printing machine commercially available has a built volume of 1000 × 800 × 500 mm, representing a built size limitation if higher parts would like to be manufactured [187]. Regarding the dimensional accuracy of the final part, this feature depends on many parameters, such as the layer thickness, nozzle diameter, part geometry, system resolution, part orientation, 3D printing parameters, material distortion, shrinkage, and warping [188]. 

The effects of shrinkage and warping could be generated by internal stresses presented during the cooling and crystallization, mainly of semicrystalline polymers. Polymer chains tend to organize to form dense crystalline regions, producing shrinkage behavior. Because of this shrinkage and warping effects, along with the lack of crystallinity control and poor adhesion to the 3D printer-built platform, semi-crystalline polymers such as PP, PET, or PS are not common in FDM applications [189,190,191]. 

**Figure 16:**
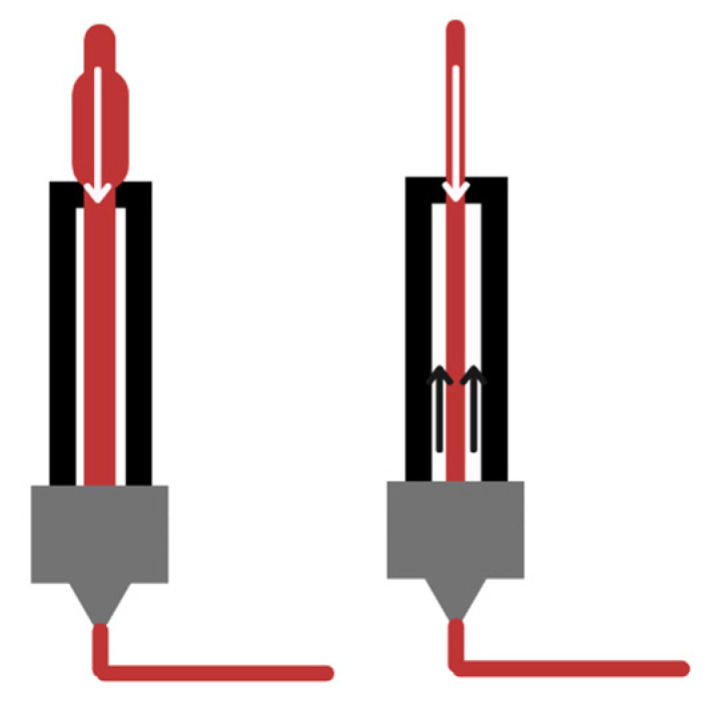
Filament inconsistencies.

In developing new FDM, a critical requirement is that feedstock materials obtain a constant cross-sectional diameter. During the 3D printing process, an extruder wheel pushes a specific volume of plastic down through the extruder and then to the nozzle. When the filament is irregular, the printer will not compensate for the difference, causing an “inconsistent extrusion”. As shown in Figure 16, if the filament increases in diameter, the material will not be able to fit in the nozzle and melt properly. A small diameter will cause the mechanism not to be able to push the filament due to the lack of tension [192]. 

#### 4.5.1. Recycled-Matrix 3D Printing Composites

Recycling is a potential solution to face environmental problems related to plastics, including many challenges. Mainly post-consumer plastics are contaminated by additives, fillers, or dyes, making material recovery options crucial, complex, and expensive [131]. Waste management challenges can be addressed in two different ways, depending on the type of country. Some countries where the waste collection and disposal systems are less developed, and some others with functional waste collection systems and focus on implementing circular economy concepts to add value to plastic waste [193]. 

An additional challenge lies in the reprocessing of plastic waste. During recycling cycles, polymers are exposed to high shear forces, elevated temperatures, UV light, catalyst residue, and water, with different thermal and mechanical procedures, causing a thermomechanical degradation. Moreover, the degradation activity can change structural and morphological properties, such as mechanical, rheological, and thermal properties [194]. Ong et al. investigated the in-house recycling of PLA wastes using a desktop filament extruder. After one recycling cycle, mechanical properties decrease due to hydrolytic degradation observed through the crystallinity percentage [195]. Multiple recycling cycles of PP decrease its molecular weight and viscosity in its plastic state, decreasing the tensile strength at break [196]. In addition, polymer blends are generally not miscible and compatible, which means that an effective waste sorting process is important to the quality of recycling products. Furthermore, plastic products are subjected to photo-oxidation processes in their lifetime, including exposure to heat, oxygen, light, radiation, moisture, and mechanical stress [197]. This conduces to oxygenated groups formation on the polymer affecting mechanical properties. 

Garmuleewicz et al. identified technical, economic, and organizational barriers to recycling local plastic waste into filament. There is still a lack of standardization of materials and quality control concerning technical barriers. Also, the absence of efficient waste management plans represents an economic barrier. The price of the recycled polymer compared to the virgin and the cost of the recycling process compared to the alternatives of waste disposal are also important economic barriers. Finally, social and organizational barriers can be identified through the lack of acceptance of recycled filaments rather than virgin ones [198]. There is a lack of knowledge about the availability of recycled plastics, their quality, and their applications, which discourages using recycled materials [199]. Furthermore, recycled plastic blends need improving interfacial adhesion by using compatibilizers, additives, or electron irradiation due to the immiscibility and incompatibility of different polymers.

#### 4.5.2. Natural Fiber 3D Printing Filaments Biocomposites

Even though studies focused on natural fiber biocomposites have increased by the year, their application to 3D printing filaments is challenging. During filament extrusion, materials are subjected to elevated temperatures, which can cause the degradation of the fibers affecting composite performance [200]. Another crucial factor during filament fabrication is the fiber content. It seems that mechanical strength is improved with fiber addition. However, a high fiber percentage in the material can lead to wetting problems and an increasing material’s brittleness [185]. Fiber weight ratio also affects unique properties of the 3D printed parts, such as the density, tensile, flexural, and structural properties [201]. Osman et al. evaluated the mechanical properties of 3D printed parts based on ABS and rice straw (RS), varying the fiber weight ratio from 5 to 15 wt.%. Tensile and flexural properties decrease as the RS weight ratio increases [158]. Kariz et al. obtained a similar result investigating the mechanical properties of PLA/wood powder for 3D printed parts [144]. In general, implementing natural fibers results in a higher melting viscosity; thus, high power would be needed for extrusion through the nozzle, making 3D printing difficult.

Consequently, the natural filler does not exceed 20–30 wt.% [201]. Continuous and discontinuous fiber addition in 3D printing filaments tends to increase porosity in printed parts, making them likely to absorb water and unsuitable to be applied in humid environments [201]. In addition, during filament manufacturing using natural fibers, the drying process is crucial to avoid hydrolytic degradation caused by remained water in the composite. 

Furthermore, using natural fiber biocomposites in the FDM technique can be arduous due to the small nozzle size and the size of fiber particles in the filament. Obtaining a homogenous mix of polymeric matrixes with natural fibers is complicated, whereby a nozzle clog could occur due to the agglomeration of the particles [202]. This behavior was observed by Petchwattana in PLA/teak wood composites when a particle size of 125 µm was used [203]. 

The incompatibility between the fibers’ hydrophilic nature and the polymers’ hydrophobia is another common challenge researchers face when natural fiber biocomposites are being developed. The joining of the two components reduces the fiber-matrix stress transfer and the mechanical properties. Also, the water absorption rate increases, causing the reduction of dimensional stability [143,158]. 

Besides, the correct management strategies, such as composting, recycling, or incineration of natural fiber biocomposites, also represent a challenge after the material lifecycle. Yang et al. reported a lack of efficient collection and transportation systems to deliver composite waste [204]. Biocomposite waste could contaminate conventional plastics during its collection, causing an effect on the quality and physical integrity of the resulting material [205]. 

Another issue about recycling and biocomposite circularity is the dismantling and separation of natural fibers from the polymeric matrix. The manufacturing processes conducted to manufacture the composite material, along with the combination of materials with different compositions, make it impossible to separate the constituents of the composite without damaging the fibers [206]. In addition, the degradation in biocomposites is complicated as the polymer, and the fiber can degrade during the recycling process. Thus, the mechanical processing of these materials leads to a decrease in the reinforcing-fiber lengths. As a result, a decrease in mechanical strength is obtained [207]. Chemical recycling has been explored; however, this alternative is not cost-effective due to the energy consumption and the long process, and it could apply just to some polymers [205].

#### 4.5.3. Colombian Challenges

Even though 3D printing technology is growing in Colombia, there is a significant gap in education and technology accessibility. According to DANE and the Colombian Ministry of Education, in 2021, 40% of children received primary education, while 3.9% of youth could access superior education [208,209]. In addition, in Colombia, although there is no data about 3D printing technologies, there are more than 20 million people without internet accessibility. In rural areas, just 17% of students have computer and internet access. Knowing about the technological situation is crucial to understand the Colombian challenges. Nowadays the Technology Ministry is developing strategies to teach the population about digital abilities, develop technological infrastructure with high capacity, and change politics according to the presented advances.

Regarding 3D printing users, two types are identified: the industrial market, where high-performance materials are required for engineering applications, and amateur creators for personal use. A challenge to implementing new materials in 3D printing in the industrial sector is the fulfillment of requirements in terms of properties for specific applications, for example, in applications where the material is subjected to high stresses. For the amateur sector, the innovation, the concept of circular economy, and the use of unique materials are attractive. However, there is a lack of knowledge about how to print new materials which are different from conventional ones.

Colombia is also working to contribute to the Sustainable Development Goals. The laws have become more restrictive on environmental issues. For example, in 2022, the law prohibiting single-use plastics was approved. This encourages the reuse and recycling of these plastic materials while applying circular economy concepts. For this reason, many companies are considering changing the material flow to avoid single-use plastics and promoting recycling or using more eco-friendly, biobased, or biodegradable materials. At this point, it is important that industry and academia join to promote the development of new materials that comply with the new regulations and prove to be more sustainable and eco-friendlier.

## 5. Conclusions

This work explored circular economy concepts applied in agro-industrial and plastic waste valorization. A literature review was performed to map and look over the potential, advances, challenges, and opportunities in recycled plastics and natural fibers as fused deposition modeling feedstock. Colombian capacity to develop natural fiber biocomposite materials with local residues due to its considerable biodiversity was also presented. Also, the importance of adopting new technologies, such as 3D printing, developing new materials in the country’s progress and accomplishing the 2030 Agenda and the Sustainable development goals were identified. 

Based on the results, though natural fiber biocomposites and recycled plastic materials are being widely studied for 3D applications, the implementation of both materials in this manufacturing technique has been less studied. Literature showed the potential of these more sustainable materials to move toward a circular economy while the environmental issue caused by landfilled waste is reduced and innovation and industry competitiveness is achieved. An opportunity to investigate 3D printed parts applications and possible solutions to the exposed challenges was also identified.

## Figures and Tables

**Table 1:** Applications of FDM technology.

Segment	Applications	Common Materials
Automotive	Engine access doors, inlet guide vanes, acoustic liners, cover plates, jigs, fixtures, check gauges, interior accessories, lights, bezels, and full-scale panels.	Polycarbonate, nylon, ULTEM
Shielding application	Conductive components, energy storage devices, sensors	Polylactic acid (PLA)/graphene electrodes, PLA/carbon nanotube/zinc oxide PLA/copper
Medical	Tissue engineering, orthopedic, biomedical applications	Polycaprolactone/tricalcium phosphate Polydopamine/PLA Polylactic-co-glycolic acid/titanium dioxide Polyethylene glycol/indomethacin
Pharmaceutical	Drug delivery devices, precision medicine	Polyvinyl alcohol Polycaprolactone
Rapid tooling	Prototypes, functional parts, customized components	PLA, Acrylonitrile butadiene styrene (ABS), polypropylene (PP)
4D prinitng	Actuators for soft robotics, controlled sequential folding systems, self-evolving structures	

Based on [77,78].

**Table 2:** Melting temperatures and some properties of different 3D printing common materials.

Polymer	Melting Temperature (°C)	Melt Mass-Flow Rate (g/10 min)	Tensile Stress at Break (MPa)	Applications
Polylactic acid (PLA)	145–160	6.1	45.6	Rapid prototyping, accurate models, containers, automotive industry (jigs/fixtures)
Acrylonitrile butadiene styrene (ABS)	225–245	41.0	33.9	Functional prototyping, concept modeling, production of tooling and end-use parts, architectural models, medical prostheses, toys
Polypropylene (PP)	130	20.0	35.1	Low strength applications, living hinges, straps, leashes.
Thermoplastic polyurethane (TPU)	220	15.9	39.0	Flexible, functional prototypes and end-use parts, sporting goods, protective cases, automotive bushings, vibration-damping components
Polyethylene terephthalate glycol (PETG)	260	6.4	38.5	Manufacturing (bottles/food containers) Packaging (pharmaceutical and medical device packaging)
Nylon	185–195	6.2	34.4	Snaps fit, living hinges, gears. Automotive and aerospace industry (jigs, prototypes for interior panels, low-heat air-intake components. Medical sector (anatomical models, end-use parts)
Polycarbonate (PC)	60	32–35	76.4	Sunglass lenses, scuba masks, electronic display screens, phone cases)

Based on [95,96,97].

**Table 3:** Natural fiber biocomposites studied in FDM applications.

Polymeric Matrix	Filler	Fiber Content (wt.%)	Main Results	Reference
PLA	Flax	34	Irregular filament. Increase in tensile strength when compared to short fibers.	[147]
PLA	Flax	-	Tensile strength was improved by 89% compared to PLA. Flexural strength increased by 211% when compared to neat PLA.	[151]
PLA	Flax	30	3D printed parts decreased mechanical properties compared to specimens manufactured by injection molding.	[152]
PLA	Jute	-	Jute continuous fiber composite exhibited an increase in tensile strength compared to neat PLA 3D printed specimens.	[149]
PLA	Hemp	-	3D printed PLA and hemp specimens presented improved tensile strength compared to the neat PLA.	[150]
PLA	Hemp Harakeke	10, 20, 30	A decrease in tensile strength was obtained with increasing fiber content. Young’s moduli tend to increase with increasing fiber content. PLA and harakeke composites increased Young’s moduli by 42.5% compared to neat PLA.	[153]
PLA	Bamboo	20	Reinforcing the PLA filament with long bamboo fibers increases the modulus by 91% compared to dust bamboo. Void content was between 0–4%.	[148]
PLA	Bamboo	12	The tensile test showed that specimens printed at 0° showed the least ductile behavior at a maximum elongation of 2.4%, while the 0°/90° was the most ductile at 5.1%.	[154]
PLA	Bamboo	20	Little adhesion between PLA and bamboo fibers, allowing their separation	[155]
PLA	Wood Rice husk	10	PLA and wood composites flexural modulus increased by 25% compared to the PLA and rice husk composites’ modulus	[156]
PLA	Cork	5, 10, 15, 20, 25, 30, 50	As cork content increase, composites’ density decrease. Specific modulus and tensile strength improved as cork content increased. Tributyl citrate was used to overcome PLA brittleness.	[157]
ABS	Rice straw	5, 10, 15, 20	Tensile and flexural properties decreased as the fiber content increased.	[158]
ABS	Macadamia nutshell	19, 29	3% of maleic anhydride was used. Density decreases as fiber content increases. Tensile strength decreases as fiber content increases.	[159]
PCL	Micronized cocoa shell	10, 20, 30, 40, 50	Tensile strain decreases as fiber content increases. With more than 40 wt.% of cocoa shells, the 3D printing process was occasionally interrupted due to the clogging of the 3D printer nozzle.	[160]

## Data Availability

Not applicable.
